# Comment on ‘Domestic light at night and breast cancer risk: a prospective analysis of 105 000 UK women in the Generations Study’

**DOI:** 10.1038/s41416-018-0058-1

**Published:** 2018-10-03

**Authors:** Richard G. Stevens

**Affiliations:** University of Connecticut, School of Medicine, 263 Farmington Avenue, Farmington, CT 06032 USA

**Keywords:** Risk factors, Scientific community

Due to massive exposure misclassification, the analysis by Johns et al.^1^ yields no evidence, one way or the other, bearing on whether light at night (LAN) raises risk of breast cancer in women. The exposure surrogate used in the study is tantamount to assigning exposure to each study subject on the basis of the flip of a coin. The exposure of actual interest is to excessive electric light during the night (anytime from sunset to sunrise) over a period of years or decades. Previous epidemiological studies of this question have specified various surrogates that are feasible to use in large studies of breast cancer causation, with each having its strengths and its limitations as estimates of the exposure of interest. The most commonly used surrogate has been occupation in night shift work, which has the advantage of being reliably ascertained from study subjects, but has the limitations of potential for confounding and the fact that day workers are also exposed to LAN, though theoretically not as much.

The entire exposure assessment protocol of the study by Johns et al.^1^ was comprised of a single subjective question: what was the relative ambient light level in the bedroom after lights out for sleep, currently and at age 20? (For the majority of subjects, the response for exposure at age 20 was many years, or decades, in the past). Of course, this exposure surrogate offered no information on light exposure during the evening, from sunset to when lights were turned out for sleep, which is typically many hours. This period is crucial for excessive light to have impact in disrupting circadian rhythmicity by delaying transition to night-time physiology, which should begin at sunset. Experimental studies in humans have shown that relatively brighter, shorter wavelength light (ie, bluer) in the evening delays this important physiological transition compared to dimmer, longer wavelength light.^2–4^ Also, once lights are out in a bedroom at night, very few people experience an ambient light level high enough to suppress melatonin under controlled laboratory conditions.

The problem of false-positive findings in epidemiological studies has received wide attention, whereas the problem of false negatives has not.^5^ Sufficiently poor exposure assessment will guarantee a null finding even if there is a strong association of the actual exposure of interest (eg, chronic, excessive electric light during the night, anytime between sunset and sunrise) and the outcome (eg, breast cancer). This study is analogous to the recent large study by Travis et al.^6^ that claimed to show no association between shift work and risk of breast cancer (which it most assuredly did not show^7^). In both studies, the exposure surrogate was virtually unrelated to the exposure of actual interest. The use of poor exposure assessment allows a large study to receive attention without advancing any understanding of disease causation.
